# Exposure of Monocytes to Lipoarabinomannan Promotes Their Differentiation into Functionally and Phenotypically Immature Macrophages

**DOI:** 10.1155/2015/984973

**Published:** 2015-08-11

**Authors:** Leslie Chávez-Galán, Ranferi Ocaña-Guzmán, Luis Torre-Bouscoulet, Carolina García-de-Alba, Isabel Sada-Ovalle

**Affiliations:** ^1^Laboratory of Integrative Immunology, National Institute of Respiratory Diseases Ismael Cosio Villegas, 14080 Mexico City, DF, Mexico; ^2^Department of Physiology, National Institute of Respiratory Diseases Ismael Cosio Villegas, 14080 Mexico City, DF, Mexico; ^3^Department of Pulmonary Fibrosis, National Institute of Respiratory Diseases Ismael Cosio Villegas, 14080 Mexico City, DF, Mexico

## Abstract

Lipoarabinomannan (LAM) is a lipid virulence factor secreted by *Mycobacterium tuberculosis* (Mtb), the etiologic agent of tuberculosis. LAM can be measured in the urine or serum of tuberculosis patients (TB-patients). Circulating monocytes are the precursor cells of alveolar macrophages and might be exposed to LAM in patients with active TB. We speculated that exposing monocytes to LAM could produce phenotypically and functionally immature macrophages. To test our hypothesis, human monocytes were stimulated with LAM (24–120 hours) and various readouts were measured. The study showed that when monocytes were exposed to LAM, the frequency of CD68^+^, CD33^+^, and CD86^+^ macrophages decreased, suggesting that monocyte differentiation into mature macrophages was affected. Regarding functionality markers, TLR2^+^ and TLR4^+^ macrophages also decreased, but the percentage of MMR^+^ expression did not change. LAM-exposed monocytes generated macrophages that were less efficient in producing proinflammatory cytokines such as TNF-*α* and IFN-*γ*; however, their phagocytic capacity was not modified. Taken together, these data indicate that LAM exposure influenced monocyte differentiation and produced poorly functional macrophages with a different phenotype. These results may help us understand how mycobacteria can limit the quality of the innate and adaptive immune responses.

## 1. Introduction

Tuberculosis (TB) is an infectious disease that is still one of the leading causes of mortality around the world. According to the World Health Organization, there were 9 million new cases of TB and 1.5 million deaths in 2013 [1].* Mycobacterium tuberculosis* (Mtb), the causal agent of TB, is transmitted through inhalation of aerosolized droplets that gain access to the pulmonary alveoli. Once there, the bacteria bind different phagocytic receptors and enter resident alveolar macrophages, dendritic cells and recruited monocytes from the bloodstream, thus developing a cellular structure called granuloma [2].

Mtb is an intracellular pathogen whose cell wall structure accounts for its low permeability and resistance to antibiotics. Its main structural components are lipids, carbohydrates and a small fraction of proteins, and molecules that play a critical role in TB pathogenesis. The mycobacterial cell envelope is multilayered with an interspace between the plasmatic membrane and the cell wall, which contains several macromolecules covalently attached, such as peptidoglycans and arabinogalactans, as well as an exterior layer of mycolic acids [3].

Scientific evidence demonstrates that mycolic acids and lipoarabinomannan (a phosphatidylinositol-derived lipoglycan, LAM), both of which are considered virulence factors, can be identified in the sputum and urine of TB-patients and, consequently, could be implemented as diagnostic biomarkers of TB, especially in countries with limited resources [4–6]. Moreover, the presence of LAM in the peripheral blood of TB-patients has recently been demonstrated by a sandwich immunoassay format and, due to its amphiphilic nature, it can also be associated with host lipid carriers [7]. These data suggest that peripheral blood mononuclear cells from TB-patients might be exposed to TB lipids (LAM) during the natural history of pulmonary TB.

Using a mouse model, Sköld and Behar demonstrated that circulating monocytes may be the direct precursors of the macrophages and dendritic cells found in Mtb-infected lungs and draining pulmonary lymph nodes in pulmonary TB [8]. However, various studies have shown that peripheral blood monocytes from TB-patients can give rise to dendritic cells that are not optimal in terms of either differentiation or maturation and that this abnormal phenotype might be responsible for both dysfunctional T cell activation and an impaired cellular immune response. Also, our group has demonstrated that circulating monocytes from TB-patients have an abnormal TLR2 and TLR4 expression profile and are more prone to presenting cell death, probably as a result of mitochondrial damage [9–11]. Based on these findings, we hypothesized that monocyte exposure to mycobacterial lipids such as LAM may alter their phenotype and function. To assess our research question we designed an* in vitro* experimental model to study the monocyte differentiation process when exposed to LAM for increasing periods of time (1–6 days). Our results provide scientific support for the notion that monocyte exposure to LAM alters the differentiation process of monocytes into macrophages; moreover, mature macrophages had an impaired function that might reduce the quality of the innate immune response against these bacteria.

## 2. Materials and Methods

### 2.1. Ethics Statement

Peripheral blood mononuclear cells (PBMCs) were acquired from buffy coats by the blood bank at the National Institute of Respiratory Diseases, Mexico City. The study was approved by the Institutional Review Board (IRB# B04-12) and was conducted following the principles stipulated in the Helsinki Declaration.

### 2.2. Lipoarabinomannan from* Mycobacterium tuberculosis* H37Rv

Purified lipoarabinomannan was obtained from the Colorado State University (NR-14848). The lipid was then reconstituted in distilled water as recommended.

### 2.3. Cells

PBMCs were isolated from buffy coats by standard Lymphoprep (Accurate Chemical-Scientific, Westbury, NY, USA) gradient centrifugation. Monocytes were isolated by positive selection using anti-CD14-coated magnetic microbeads (Miltenyi Biotech). Enrichment of the CD14^+^ fraction was routinely >95%, as analyzed by flow cytometry. CD14^+^ cells were plated at 1 × 10^6^ cells/well in 24-well plates (Costar, Ontario, Canada) with RPMI 1640 medium (GIBCO, Grand Island, NY, USA) and supplemented with L-glutamine (2 mM; GIBCO, Grand Island, NY, USA), streptomycin, penicillin, and 10% heat-inactivated fetal bovine serum (GIBCO, Grand Island, NY, USA). The CD14^+^ cells were cultured for 7 days at 37°C in a humidified atmosphere containing 5% CO_2_. After 7 days, viable cells were considered to be monocyte-derived macrophages (MDM) based on their expression profile of differentiation molecules.

### 2.4. Differentiation and Stimulation of MDM with LAM

**Figure 1:**
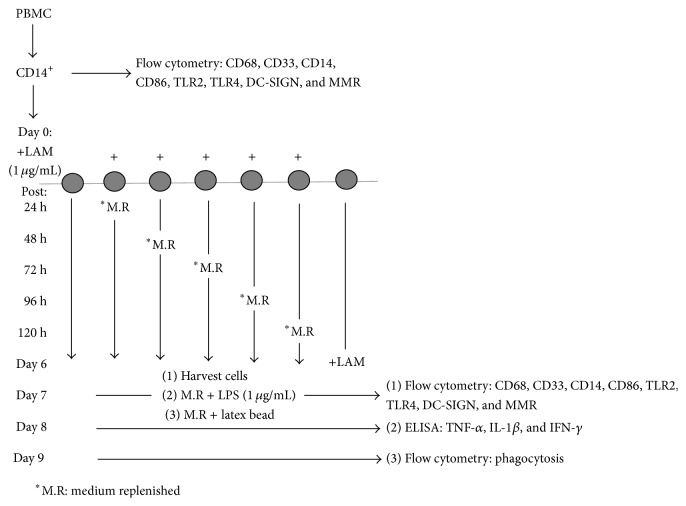
Design of the experimental strategy.

It has been demonstrated previously that LAM can be used in cell cultures for more than 24 h at a concentration of 1 *μ*g/mL, since higher concentrations are used to stimulate cells for less than 24 h [12, 13]. Thus, CD14^+^ (1 × 10^6^) cells were seeded in a 24-well plate and on day 1 were stimulated with LAM (1 *μ*g/mL). The stimulus was maintained for 24, 48, 72, 96, and 120 h. Additionally, we stimulated MDM on day 6 for 24 h. Every 24 h, the culture media were replaced in specific wells and new media were added for the rest of the incubation period. At day 7 after stimulation we established three different readouts: (1) recovering cells for FACS analysis of cell-surface markers, (2) cytokine secretion after cell stimulation for an additional 24 h with LPS (1 *μ*g/mL), and (3) phagocytic activity using cells incubated for 48 h with latex beads (Figure 1).

### 2.5. Flow Cytometry

Next, we evaluated the phenotypic profile of the exposed MDM. Briefly, cells were stained for 20 min at 4°C with fluorochrome-conjugated mAb against CD68, CD33, CD86, TLR2, TLR4, Dendritic Cell-Specific ICAM-3-grabbing nonintegrin (DC-SIGN or CD209), and the Macrophage Mannose Receptor (MMR or CD206) (BioLegend, San Diego, CA). After incubation, cells were washed and resuspended in staining buffer (BD Biosciences, San Jose, CA) prior to FACS analysis. Data were collected using a FACS Aria II flow cytometer (Becton Dickinson, San Jose, CA) and FACS Diva software (V.6.1); then the cells were analyzed with FlowJo (Tree Star, Inc. Ashland, OR). Typically, 20,000 events were acquired.

### 2.6. Latex Bead Phagocytosis

To measure phagocytosis, a latex bead assay was used in accordance with the manufacturer's instructions (Cayman Chemical Company, Ann Arbor, MI). Briefly, 1 × 10^5^ MDM were plated and exposed to LAM, as described previously in our experimental protocol. On day 7, the medium was replaced and the MDM were incubated for an additional 48 h with the latex beads-Rabbit IgG-FITC complex. After incubation, the cells were harvested and suspended in assay buffer. Data were collected using a FACS Aria II flow cytometer.

### 2.7. Cytokine Production

After the MDM were exposed to LAM, cell culture supernatants were recovered and then stored at −80°C for future analysis. We used the standard sandwich ELISA for IL-1*β*, TNF-*α*, and IFN-*γ* production following the manufacturer's instructions (BioLegend, San Diego, CA). All cytokines in the culture supernatants were quantified by comparison with the appropriate recombinant standard.

### 2.8. Statistical Analysis

Data are shown as medians and interquartile ranges (IQR). Mann-Whitney tests were used to compare two groups and a Kruskal-Wallis test with a Dunn* post hoc* test when more than two groups were compared. Values of *P* < 0.05 were considered statistically significant (GraphPad Software, Inc., San Diego, CA).

## 3. Results

### 3.1. Phenotypic Characterization of Monocyte-Derived Macrophages Generated* In Vitro*

**Figure 2:**
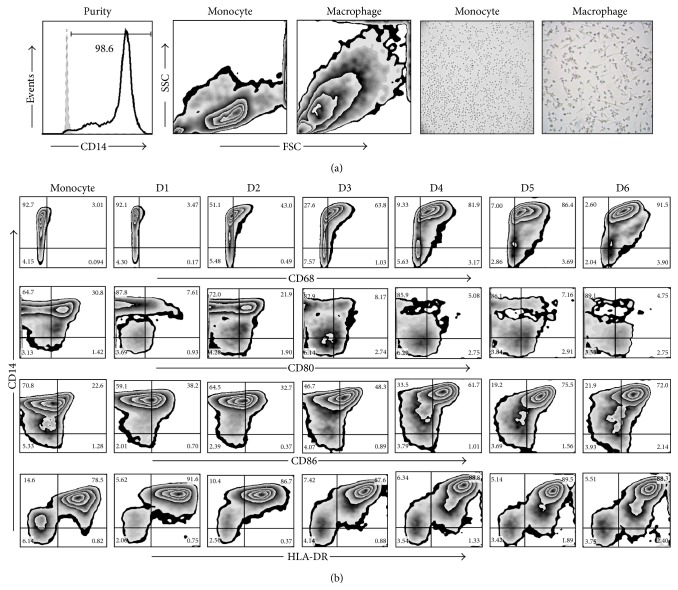
Characterization of monocyte-derived macrophages generated* in vitro*. Representative images of the purity, size, and complexity of monocytes and mature MDM by flow cytometry and morphology by microscopy (a). Expression of CD68, CD80, CD86, and HLA-DR was analyzed on a daily basis (D) by flow cytometry (b). Data are representative of one of five independent experiments.

The average purity of the enriched monocytes in all our experiments was at least 95% (Figure 2(a)). After 7 days in culture with complete RPMI-1640 the monocytes were differentiated into mature macrophages [14]. The mature phenotype was confirmed by several parameters, such as changes in size, granularity, and cell-surface expression of CD68, CD80, CD86, and HLA-DR, all of which were analyzed by flow cytometry (Figures 2(a) and 2(b)).

### 3.2. Production of Mature Macrophages Is Limited When Monocytes Are Exposed to LAM

**Figure 3:**
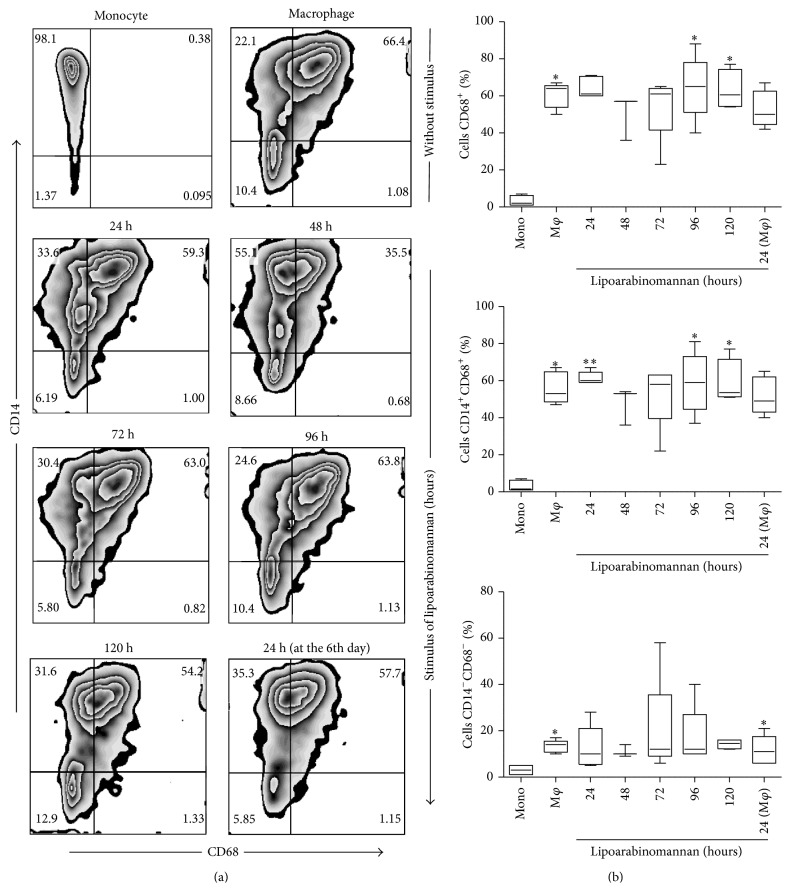
Frequency of CD14^+^CD68^+^ macrophages decreases when monocytes are exposed to LAM. Pure monocytes were incubated with LAM for 24–120 h at 37°C. All cells were recovered on day 7. Representative zebra plots for CD68 versus CD14 (a). A percentage of CD68^+^, CD14^+^CD68^+^, and CD14^−^CD68^−^ are shown. Data are representative of five independent experiments. Box plot indicates median ± IQR (5–95). ^*∗*^ *P* < 0.05, ^*∗∗*^ *P* < 0.01. Kruskal-Wallis and Dunn* post hoc* tests compared to unexposed monocytes.

**Figure 4:**
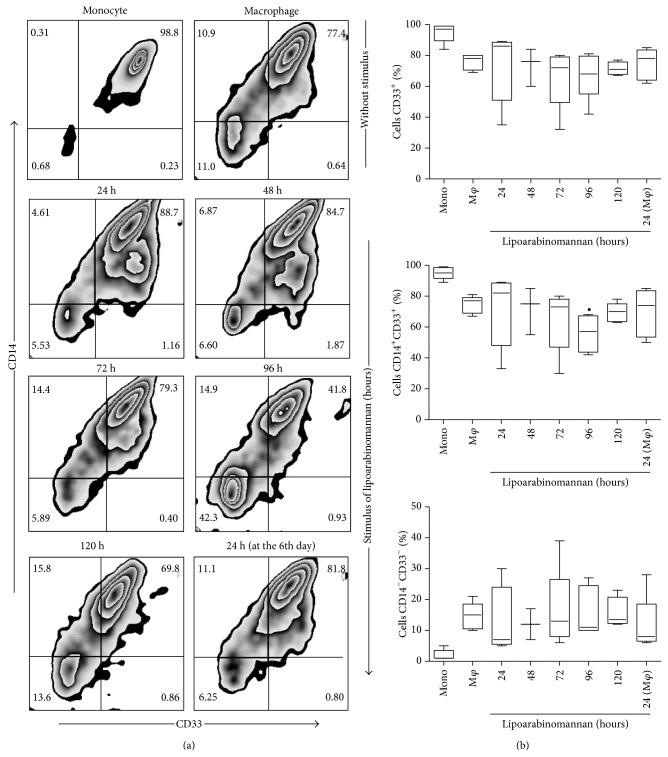
The percentage of macrophages CD14^+^CD33^+^ decreases when monocytes are exposed to LAM. CD14^+^ monocytes were incubated with LAM for 24–120 h at 37°C. Representative zebra plots for CD33 versus CD14 (a). A percentage of CD33^+^, CD14^+^CD33^+^, and CD14^−^CD33^−^ are shown. Data are representative of five independent experiments. Box plot indicates median ± IQR (5–95). ^∙^ *P* < 0.05. Kruskal-Wallis and Dunn* post hoc* tests compared to unexposed MDM.

**Figure 5:**
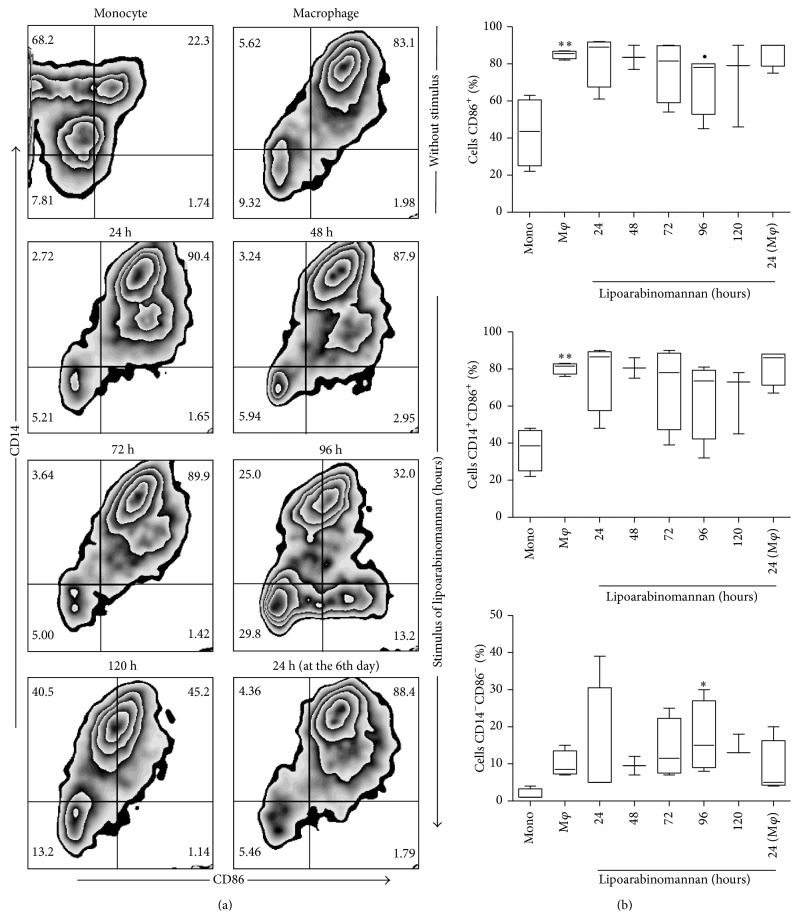
The percentage of macrophages CD86^+^ decreases when monocytes are exposed to LAM. CD14^+^ monocytes were incubated with LAM for 24–120 h at 37°C. Representative zebra plots for CD86 versus CD14 (a). A percentage of CD86^+^, CD14^+^CD86^+^, and CD14^−^CD86^−^ are shown. Data are representative of five independent experiments. Box plot indicates median ± IQR (5–95). ^∙^ *P* < 0.05, ^*∗∗*^ *P* < 0.01. Kruskal-Wallis and Dunn* post hoc* tests compared to unexposed monocytes (*∗∗*) or to unexposed MDM (∙).

Our first approach was to analyze whether monocyte exposure to LAM would alter the mature phenotype of the macrophages. We ascertained that when monocytes were exposed to LAM for increasing periods of time, different macrophage subsets were obtained based on CD14 and CD68 expression profiles (Figure 3(a)). Total CD68 expression on unexposed mature macrophages was significantly higher than that observed on fresh monocytes, a result that is consistent with previous reports [15]; however, it appears that when monocytes were exposed to LAM for 48-to-72 h, the cells tended to reduce CD68 expression (Figure 3(b)), though this result did not reach statistical significance. We also included the analysis of CD33 and CD86 markers due to their importance as monocyte differentiation markers [16, 17], observing that when monocytes were exposed to LAM for 96 h, the frequency of CD33^+^ and CD86^+^ cells tended to decrease. However, statistically significant differences were not identified (Figures 4 and 5). On the basis of these data we speculate that when the monocytes were exposed to a microenvironment in which LAM was present, slight macrophage phenotypic changes were induced. Each molecule has a specific expression profile, but this may be altered in a time- and concentration-dependent manner when cells are exposed to LAM. We found that, between 48 and 72 h of LAM exposure, CD68 expression showed a discrete reduction, while both CD33 and CD86 expression decreased at 96 h. Thus, we hypothesized that in TB-patients peripheral blood monocytes are constantly exposed to LAM for unknown periods of time, and this may alter their differentiation and, potentially, their function.

### 3.3. TLR2 and TLR4 Expression Is Altered When Monocytes Are Exposed to LAM

**Figure 6:**
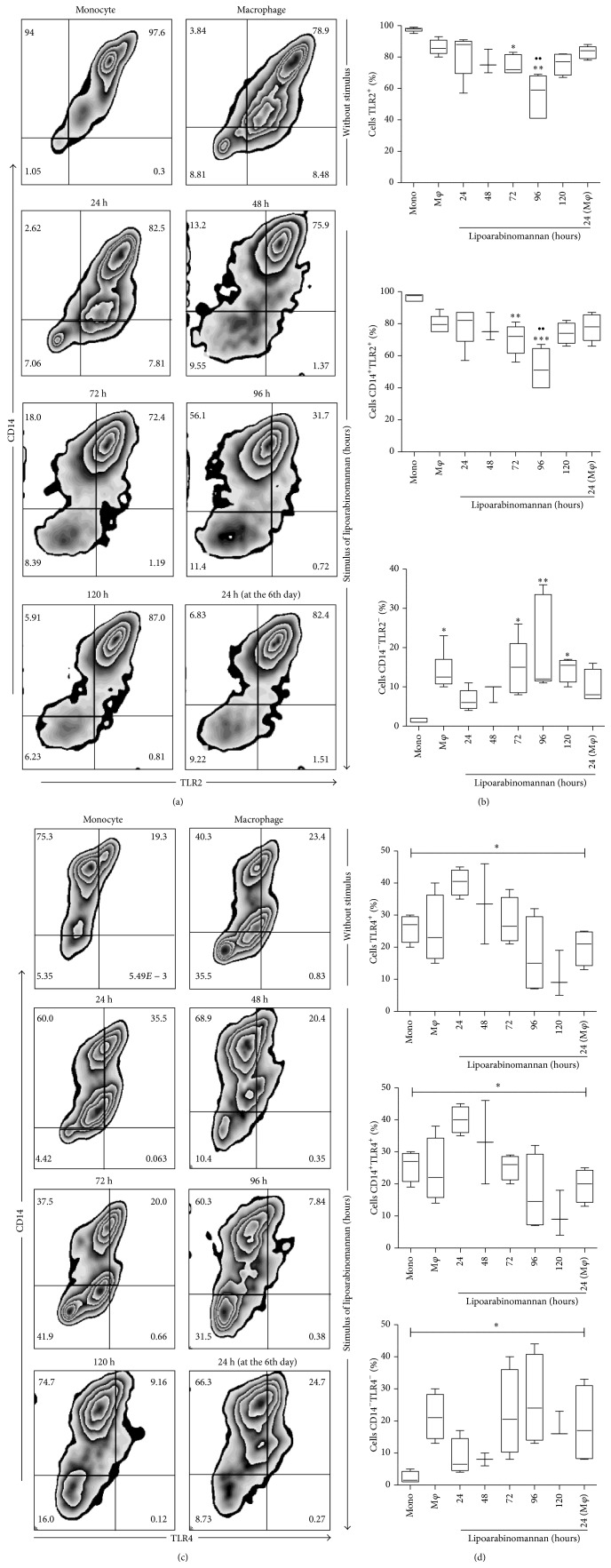
The percentage of macrophages TLR2^+^ and TLR4^+^ decreases when monocytes are exposed to LAM. CD14^+^ monocytes were incubated with LAM for 24–120 h at 37°C. Representative zebra plots for TLR2 and TLR4 versus CD14 ((a) and (c)). A percentage of TLR2^+^, CD14^+^TLR2^+^, CD14^−^TLR2^−^, TLR4^+^, CD14^+^TLR4^+^, and CD14^−^TLR4^−^ are shown. Data are representative of five independent experiments. Box plot indicates median ± IQR (5–95). ^*∗*^ *P* < 0.05, ^*∗∗*^ *P* < 0.01. Kruskal-Wallis and Dunn* post hoc* tests compared to unexposed MDM.

TLR2 and TLR4 play a central role in the immune response against tuberculosis by secreting proinflammatory cytokines and activating other molecular mechanisms, such as autophagy, which can limit intracellular bacterial growth [18–20]. To determine whether monocyte exposure to LAM could change the expression profile of molecules that are important for macrophage activation and functionality, TLR2 and TLR4 expression was evaluated, with the result that the frequency of TLR2^+^ macrophages did not differ from that observed in fresh monocytes; however, when monocytes were cultured in the presence of LAM, the percentage of TLR2^+^ macrophages declined compared to both unexposed monocytes and macrophages (Figure 6). These data show that the percentage of macrophages that express CD14 and TLR2 decreased under LAM stimulation and that this was proportional to the increased percentage of double-negative cells (CD14^−^TLR2^−^). A similar phenomenon was observed with TLR4 expression (Figure 6(e)), though the loss of TLR4 was less dramatic and required more exposure time than LAM (120 h). The TLR2 and TLR4 expression profile observed in mature macrophages seems to have a bimodal pattern; unfortunately, there is no evidence of how mycobacterial lipids modulate the kinetics of TLR2 and 4 expression when analyzed in an* in vitro* experimental model similar to the one proposed herein. The possibility that abnormal monocyte/macrophage differentiation is related to the impaired expression of these markers cannot be ruled out.

**Figure 7:**
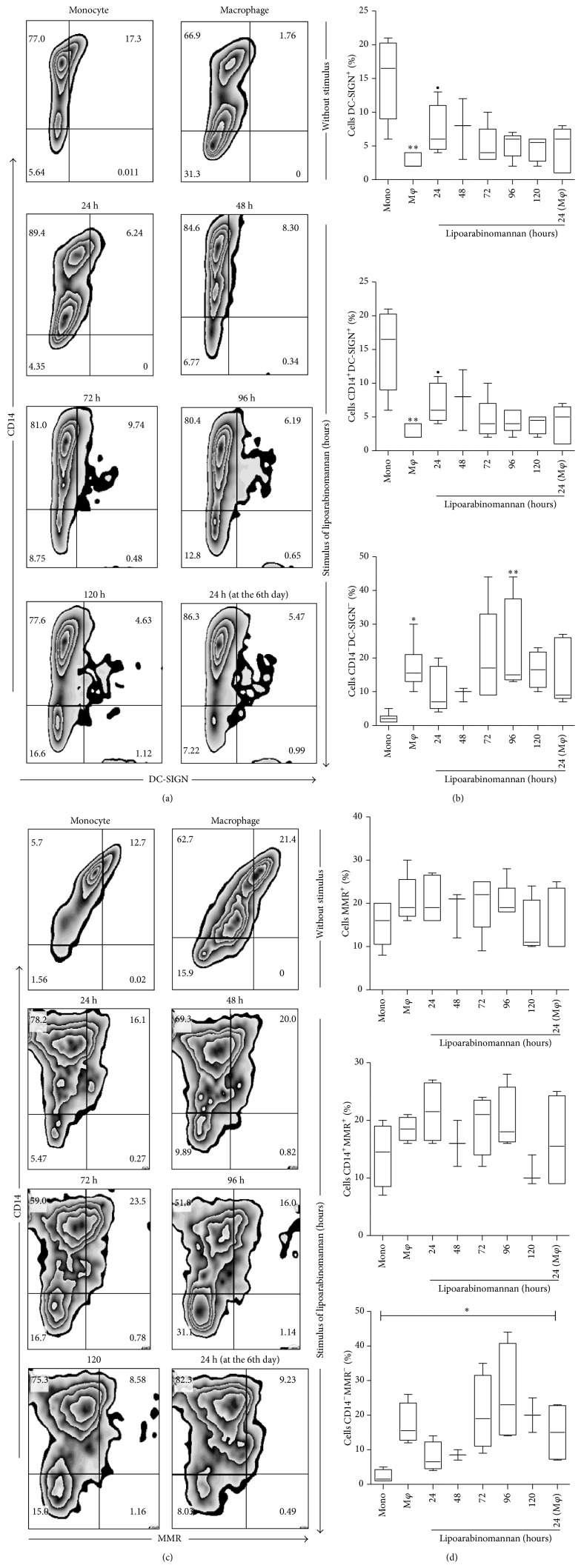
The percentage of macrophages DC-SIGN^+^, but not MMR^+^, increases when monocytes are exposed to LAM. CD14^+^ monocytes were incubated with LAM for 24–120 h at 37°C. Representative zebra plots for DC-SING and MMR versus CD14 ((a) and (c)). Percentages of DC-SIGN^+^, CD14^+^DC-SIGN^+^, CD14^−^DC-SIGN^−^, MMR^+^, CD14^+^MMR^+^, and CD14^−^MMR^−^ are shown. Data are representative of five independent experiments. Box plot indicates median ± IQR (5–95). ^∙^ *P* < 0.05, ^*∗*^ *P* < 0.05, and ^*∗∗*^ *P* < 0.01. Kruskal-Wallis and Dunn* post hoc* tests compared to unexposed monocytes (*∗∗*) or to unexposed MDM (∙).

C-type lectins are a family of soluble, surface-bound receptors that are essential for pathogen recognition. DC-SIGN (Dendritic Cell-Specific Intercellular adhesion molecule-3-Grabbing Nonintegrin) and MMR (Macrophage Mannose Receptor) are among the most important receptors in this family that participate in pathogen interaction through mannose and fucose recognition [21]. DC-SIGN is expressed by dendritic cells (DC) and by a small percentage of monocytes and M2 macrophages [22, 23]. This study found that less than 5% of untreated macrophages expressed DC-SING, compared to 15% of monocytes (Figure 7(b)). When monocytes were exposed to LAM for 24 h, the frequency of DC-SIGN^+^ macrophages was significantly higher than that observed in unexposed macrophages. The frequency of DC-SIGN^+^ macrophages generated after only 24 h of LAM exposure tended to diminish over time, suggesting that DC-SIGN expression on the macrophage is influenced by the cell microenvironment. This is important because not all macrophages are exposed to Mtb lipids for the same amount of time. Regarding MMR expression, results showed that exposure to LAM did not affect the expression of this molecule on macrophages (Figure 7(d)). All these data lead to the suggestion that different macrophage subsets were produced when monocytes were exposed to LAM. A variable phenotypic profile might be associated with changes in macrophage function that can impact their ability to control intracellular bacterial growth. Molecules such as TLR4 and DC-SIGN seem to be affected after only 24 h of exposure to LAM, while TLR2 expression decreases when monocytes are exposed for 72-to-96 h.

### 3.4. Monocytes Exposed to LAM Are Differentiated into Dysfunctional Macrophages

**Figure 8:**
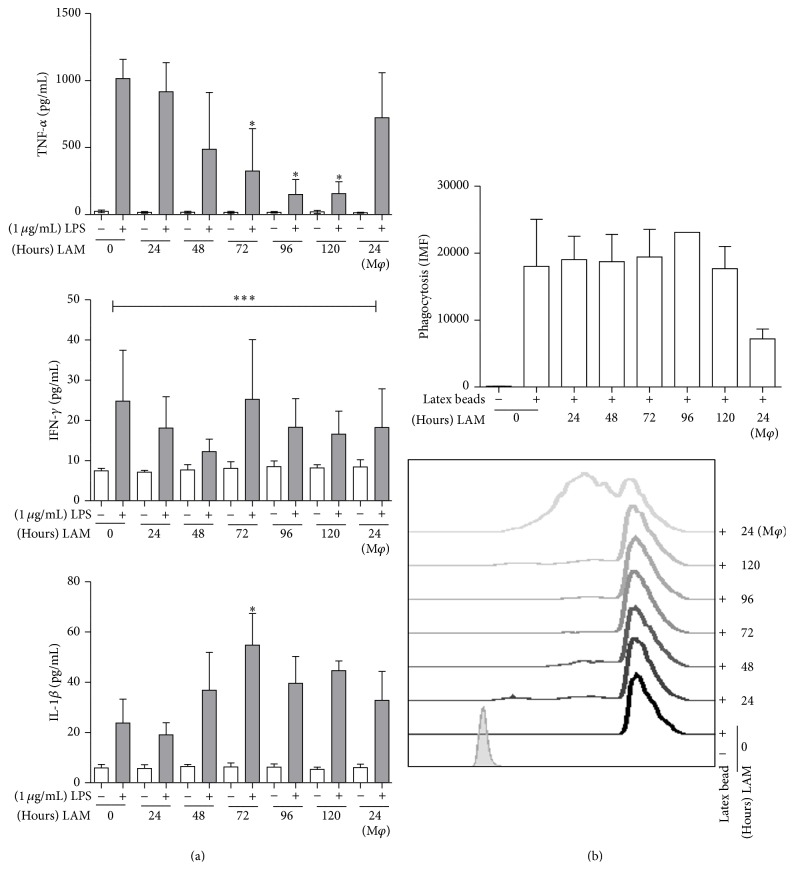
Monocytes exposed to LAM are differentiated into functionally deficient macrophages. CD14^+^ monocytes incubated with LAM for 24–120 h at 37°C. On day 7 cells were stimulated with LPS (1 *μ*g/mL) for 24 h. TNF*α*, IL-1*β*, and IFN-*γ* in the supernatant were measured by ELISA (a). Phagocytosis of latex beads was analyzed on the exposed MDM. Data are representative of five independent experiments in (a) or two independent experiments in (b). Box plot indicates median ± IQR (5–95). ^*∗*^ *P* < 0.05, ^*∗∗∗*^ *P* < 0.001. Kruskal-Wallis and Dunn* post hoc* tests compared to unexposed MDM.

Based on previous results and considering their potential implication in macrophage antibacterial activity, the decision was taken to evaluate the production of proinflammatory cytokines, such as tumor necrosis factor-alpha (TNF*α*), interleukin 1beta (IL-1*β*), and Interferon-gamma (IFN-*γ*). Also evaluated was the phagocytic capacity of the macrophages that were derived from monocytes exposed to LAM. Results showed that when the monocytes were stimulated with LAM for 72-to-120 h, mature macrophages released less TNF-*α* than the macrophages cultured without LAM or those exposed to LAM for shorter times (24–48 h). No significant reduction in IFN-*γ* secretion was observed, and the highest concentration was identified when IL-1*β* was analyzed after 72 h of LAM stimulation (Figure 8(a)).

Phagocytosis by activated macrophages is one of the most important macrophage functions due to its impact during immunity against intracellular pathogens, such as Mtb. To evaluate whether this function was conserved in macrophages exposed to LAM, a phagocytosis assay was used. Results showed that when monocytes were exposed for longer time periods, phagocytosis in mature macrophages was not affected; however, when mature macrophages were exposed to the final 24 h of LAM, phagocytosis decreased by nearly 50% (Figure 8(b)).

Together, these results demonstrate that if monocytes are exposed to a microenvironment containing an Mtb lipid like LAM, generation of a mature phenotype might be modified and functionality reduced. Both phenotypic and functional alterations were time-dependent on exposure to LAM.

## 4. Discussion

Current knowledge of the process of differentiation from monocytes to macrophages or dendritic cells (DC) is well-established; however, there is evidence showing that monocytes from TB-patients differentiate into DC that are incapable of inducing an efficient immune response. These monocytes are more susceptible to cell death and show phenotypic alterations when compared to monocytes from healthy donors [10, 11, 24]. Recently, it was demonstrated that serum from TB-patients may contain some components of the mycobacterial cell wall, such as LAM (glycolipid nature), which can potentially induce an inadequate process of differentiation from monocyte to DC [7, 25]. Based on a previous publication by our group which demonstrated that monocytes from TB-patients are more prone to presenting cell death and considering that monocytes are temporarily exposed to a microenvironment with LAM, we hypothesized that their differentiation process might be altered and could lead to the production of immature macrophages that are less capable of controlling bacterial growth.

Thus, the main findings of this study are as follows: (1) generation of mature macrophages decreases when monocytes are exposed to LAM, (2) TLR2 and TLR4 expression is altered when monocytes are exposed to LAM, and (3) LAM-exposed monocytes differentiate into dysfunctional macrophages.

In order to evaluate macrophage function we designed an* in vitro* experimental system that allowed us to analyze monocyte differentiation under various experimental conditions. After 7 days in culture, we obtained macrophages that were mature according to their phenotypic profile [26, 27]. To answer the first question concerning the potential impact of LAM on monocyte differentiation, we exposed monocytes for increasing periods of time, from 24 to 120 h, or only during the final 24 h of culture. Phenotypic markers such as CD33, CD68, and CD86 reduced their expression at different time-points compared to unexposed macrophages (Figure 3). Similar to previously published results the study identified that CD33 expression is high in monocytes but undergoes a small reduction when differentiated into mature macrophages [28, 29]. Monocyte exposure to LAM reduced CD33 expression more at the 72–96 h time-point; however, no statistically significant differences were found. Previously, Castaño et al. showed that whole Mtb could interfere with the process of monocyte-to-macrophage differentiation based on CD68 and CD86 expression profiles, while a similar result was found on DC differentiation and maturation [30]. It is important to emphasize that this study obtained similar results using pure LAM, not the entire bacteria, as we were able to show that exposure to this lipid during 48–72 hrs was sufficient to lower the frequency of CD68^+^ MDM. This percentage was similar to that measured on monocytes with only 3 days of differentiation (Figure 2). When markers associated with maturation were analyzed, such as CD33 and CD86 (Figures 4 and 5), it appeared that a longer time period was required to change their expression profiles. Contrasting results with respect to the effect of LAM on DC maturation and differentiation have been published previously. Mazurek et al., for example, indicated that LAM helps activate DC and that phosphatidylinositol mannosides inhibit DC activation. In contrast, Geijtenbeek et al. demonstrated that when LAM is bound to the DC-SIGN receptor, it prevents DC maturation [25, 31]. The experimental system employed in the present study showed that LAM deregulates monocyte differentiation, so it is possible that the different results on monocyte differentiation reported by previous authors are dependent on bacteria strain or cell type.

As has been described, macrophages may express different profiles and functions that are necessary to preserve the host's immunity. Two principle functions are cytokine production and phagocytosis [32]. Families of TLRs and C-type lectin receptors are essential for macrophages to function normally. TLR2 and TLR4 are two receptors that participate in immunity against Mtb by mediating the secretion of proinflammatory cytokines [18, 20, 33, 34]. Here, TLR2 and TLR4 expression on MDMs exposed to LAM were analyzed during differentiation and observation showed that when monocytes were exposed to LAM for 96 h, the frequency of TLR2^+^ macrophages was reduced compared to both monocytes and macrophages that had never been in contact with LAM (Figure 6(b)). Similar results were obtained when TLR4 expression was analyzed, but the time period required to see a reduction in TLR4 was longer (120 h) (Figure 6(e)).

After demonstrating that monocytes exposed to LAM decreased the frequency of TLR2^+^ and TLR4^+^ macrophages, we speculated that their ability to produce proinflammatory cytokines might also be abnormal. This part of the study showed that exposure to LAM impacts the macrophages' ability to secrete cytokines (Figure 8(a)), as each cytokine had a distinct time-point at which the expression profile might change; that is, macrophages exposed for 72-to-120 h decreased TNF*α* production, but IFN-*γ* began to decline after just 48 hrs. TNF*α* is a cytokine that is necessary for protection against Mtb infection. It has been demonstrated that patients undergoing treatment with anti-human TNF*α* monoclonal antibodies can reactivate latent tuberculosis, which proves the relevance of this cytokine to antimycobacterial immunity [35, 36]. Soluble TNF is cleaved by a metalloprotease-named TNF*α*-converting enzyme (TACE), and TNF*α* receptors 1 and 2 are expressed on various immune cell types and are responsible for most of the immunologic actions of this cytokine. Although the phenotypic expression profile of these molecules was not evaluated (TACE or TNF receptors), we speculate that these macrophages, generated from monocytes exposed to LAM, lack the adequate ability to secrete TNF*α* and that this phenomenon might be detrimental to controlling intracellular bacterial growth [37, 38]. Regarding IFN-*γ*, it is a critical cytokine necessary to activate and induce antimycobacterial mechanisms in macrophages. Although T cells are the major source of IFN-*γ*, human macrophages can also produce it [39, 40]. Under the present experimental conditions, it was clear that macrophages exposed to LAM for 48 h lost their ability to produce this cytokine (Figure 8(a)); however, this reduction in IFN-*γ* was transitory because at the 72 h time-point exposed macrophages recovered their ability to produce the cytokine at the same level as unexposed macrophages. These results concord with those observed by Pai et al., who showed that the 19-kDa Mtb-protein inhibited expression of the IFN-*γ* genes [41].

Contrary to our previous results with TNF*α* and IFN-*γ*, macrophages exposed to LAM for 72 h increased IL-1*β* secretion (Figure 8(a)). Macrophage TLR-dependent activation leads to IL-1*β* production through two consecutive steps: first, pro-IL-1*β* synthesis begins after a pattern recognition receptor binds to its ligand, and, second, inflammasome-activated caspase-1 drives the proteolytic processing of pro-IL-1*β* [42, 43]. Our experimental model revealed a reduction in the frequency of TLR2^+^ and TLR4^+^ macrophages after LAM exposure. If the production of active IL-1*β* occurs in response to both pathogens and damaged signals, then the deduction would be that an increased concentration of IL-1*β* could be the expected consequence of LAM exposure. LAM may generate danger signals and, by activating inflammasome, lead to increased IL-1*β* production.

One of the main functions of the macrophage is phagocytosis, which allows it to control or eliminate pathogens and activate T cells. DC-SIGN and MMR are two key receptors that mediate this function. We observed that monocyte exposure to LAM for 24 h increased the percentage of DC-SIGN^+^ macrophages (Figure 7(b)). In this expression profile, cells are more similar to monocytes than to mature macrophages. The percentage of MMR^+^ macrophages was not modified as a result of exposure to LAM (Figure 7(e)). To clarify whether phagocytic capacity was reduced, a flow cytometry assay with latex-FITC beads was used (Figure 8(b)). Data showed that the macrophages' ability to phagocyte was not modified when cells were exposed for different time periods; however, when mature macrophages were exposed to LAM for 24 h, a reduction in this capacity was observed.

In conclusion, these data demonstrate that when monocytes are exposed to a microenvironment in which LAM is present, they generate macrophages with a different phenotype that are not entirely functional. It is possible that wide varieties of cell populations will be present at the moment of recruitment into the lung and may influence the quality of the innate and adaptive immune response to Mtb. We consider that a similar phenotype might be present for resident lung macrophages and thus contribute to a defective response against the bacilli. More studies will be required to identify whether this nonfunctional phenotype is also present when monocytes are exposed to other virulence factors, such as ESAT-6 and CFP-10.
